# People living with HIV travel farther to access healthcare: a population-based geographic analysis from rural Uganda

**DOI:** 10.7448/IAS.19.1.20171

**Published:** 2016-02-10

**Authors:** Adam N Akullian, Aggrey Mukose, Gillian A Levine, Joseph B Babigumira

**Affiliations:** 1 Institute for Disease Modeling, Bellevue, WA, USA; 2 Department of Epidemiology and Biostatistics, School of Public Health, College of Health Sciences, Makerere University, Kampala, Uganda; 3 Department of Epidemiology, School of Public Health and Community Medicine, University of Washington, Seattle, WA, USA; 4 Global Medicines Program, Department of Global Health, University of Washington, Seattle, WA, USA

**Keywords:** health systems, access to HIV services, HIV antiretroviral drugs, Uganda, spatial analysis, geographic information systems

## Abstract

**Introduction:**

The availability of specialized HIV services is limited in rural areas of sub-Saharan Africa where the need is the greatest. Where HIV services are available, people living with HIV (PLHIV) must overcome large geographic, economic and social barriers to access healthcare. The objective of this study was to understand the unique barriers PLHIV face when accessing healthcare compared with those not living with HIV in a rural area of sub-Saharan Africa with limited availability of healthcare infrastructure.

**Methods:**

We conducted a population-based cross-sectional study of 447 heads of household on Bugala Island, Uganda. Multiple linear regression models were used to compare travel time, cost and distance to access healthcare, and log binomial models were used to test for associations between HIV status and access to nearby health services.

**Results:**

PLHIV travelled an additional 1.9 km (95% CI (0.6, 3.2 km), *p*=0.004) to access healthcare compared with those not living with HIV, and they were 56% less likely to access healthcare at the nearest health facility to their residence, so long as that facility lacked antiretroviral therapy (ART) services (aRR=0.44, 95% CI (0.24 to 0.83), *p*=0.011). We found no evidence that PLHIV travelled further for care if the nearest facility supplies ART services (aRR=0.95, 95% CI (0.86 to 1.05), *p*=0.328). Among those who reported uptake of care at one of two facilities on the island that provides ART (81% of PLHIV and 68% of HIV-negative individuals), PLHIV tended to seek care at a higher tiered facility that provides ART, even when this facility was not their closest facility (30% of PLHIV travelled further than the closest ART facility compared with 16% of HIV-negative individuals), and travelled an additional 2.2 km (*p*=0.001) to access that facility, relative to HIV-negative individuals (aRR=1.91, 95% CI (1.00 to 3.65), *p*=0.05). Among PLHIV, residential distance was associated with access to facilities providing ART (RR=0.78, 95% CI (0.61 to 0.99), *p*=0.044, comparing residential distances of 3–5 km to 0–2 km; RR=0.71, 95% CI (0.58 to 0.87), *p*=0.001, comparing residential distances of 6–10 km to 0–2 km).

**Conclusions:**

PLHIV travel longer distances for care, a phenomenon that may be driven by both the limited availability of specialized HIV services and preference for higher tiered facilities.

## Introduction

Rural regions of sub-Saharan Africa have some of the highest rates of HIV globally, yet the availability of specialized HIV services in those areas is often sparse or non-existent. In Africa, of the 21.2 million individuals eligible for antiretroviral therapy (ART) in 2013 under the 2013 World Health Organization (WHO) guidelines, only 7.6 million (36%) actually received ART [1]. Limited access to quality healthcare services is one of the greatest barriers to entry into the healthcare system, hindering HIV testing, treatment and care in low-resource settings [2]. HIV testing and treatment services in rural areas of sub-Saharan Africa are often limited and sparsely distributed, hindering their accessibility to the population in need [3]. Even where specialized HIV services do exist, people living with HIV (PLHIV) are faced with large economic, geographic and social barriers to access those services. Lack of information about ART, high perceived costs of the drugs, HIV stigma, inefficiencies in the healthcare system and geographic distance are among the most commonly cited barriers to accessing ART services among PLHIV [2–5]. The opportunity to reduce the burden of HIV diminishes when the economic, geographic and social costs associated with accessing healthcare outweigh their benefits [6]. Improving the availability of, and access to, high-quality, comprehensive HIV care and treatment is thus an essential part of reducing the morbidity and mortality due to HIV.

Geographic distance from residence to health facility is a major barrier to receiving adequate healthcare in sub-Saharan Africa. Individuals who reside closer to a health facility are more likely to seek healthcare than those who live farther away [7–9], and those with easier access to healthcare often have better health outcomes [10,11]. The relationship between distance and healthcare uptake has been well described for a variety of health conditions and is especially pronounced in poor, rural communities where individuals face high opportunity costs associated with accessing healthcare, such as cost of transportation and lost work time [2]. In a rural area of Kenya, for example, healthcare service use declined by 18% for each additional 0.5 km distance between an individual's residence and the primary clinic [12]. Healthcare uptake at licensed health facilities in a rural area of Uganda among children under the age of five years with febrile illness was lower for those who resided more than 3 km from the nearest health facility [13]. In a rural area of Zambia, the uptake of adequate antenatal care among pregnant women was lower among those residing farther from available health facilities [14]. In many rural areas of Uganda, economic and geographic barriers limit the uptake of HIV testing and treatment [4,15–17]. For example, pregnant women enrolled in antenatal care who resided more than 3 km from the nearest health facility with onsite HIV testing were less likely to be tested for HIV compared with those who lived closer to the clinic, leading to missed opportunities for linkage to prevention of mother-to-child transmission (PMTCT) services [16]. High cost of transport and distance are often cited as major impediments to ART uptake, adherence and continued engagement in the HIV care cascade [15,17–19].

For PLHIV, the relationship between geographic distance and healthcare access is less clear. Though PLHIV often face large economic, social and geographic barriers to accessing healthcare [20–22], in some instances, PLHIV may go farther than necessary for care [21]. The reasons why PLHIV may travel the additional distance for care are not well described. Fear of stigmatization, should they be seen and recognized by members of their community, may be one explanation for why PLHIV travel farther [23]. Stigma is a well-recognized barrier to HIV care engagement and retention and ART adherence [2,19,24–26]. Moreover, PLHIV may travel further either because they require specialized HIV services like ART, which may only be available at larger, centralized facilities providing tertiary or higher level care, which are often located in urban centres, or because they perceive those facilities to provide more comprehensive or higher-quality care.

We compared time, cost and distance of travel to access healthcare between PLHIV and those not living with HIV in a rural area of Uganda with one of the largest burdens of HIV in the country. We tested whether PLHIV are less likely than those not living with HIV to access the nearest (and often most easily accessible) health facility to their residence. We explored whether the limited availability of higher-quality services explains any differences in distance travelled to access healthcare. We secondarily tested whether PLHIV who reported having accessed a facility that provides ART tended to access the closest facility to their residence or whether they opted for a facility further away. Finally, we tested whether geographic distance to health facilities providing ART services limits the uptake of those services among PLHIV. Results of this study provide important first steps in our understanding of how to improve access to specialized HIV services among PLHIV in rural areas with limited availability of healthcare services, considering the unique geographic, economic and social constraints PLHIV face when accessing healthcare.

## Methods

### Study setting

Uganda has a generalized HIV epidemic with an adult prevalence of 6.4% and a child prevalence of 0.9% [27]. There are 1.1 million HIV-positive individuals for a population of about 30 million and incidence and prevalence have stopped declining [28]. Like many other countries in East Africa, the burden of HIV is geographically heterogeneous, with the highest prevalence in rural, coastal areas of Lake Victoria.

The study was conducted on Bugala Island in the Kalangala district of southern Uganda, the largest and the most populated of the Ssese Islands in Lake Victoria. Bugala Island has a population of approximately 58,100 people (60% male and 40% female) and is part of 84 islands of diverse geographies and populations [29]. The main economic activities involve fish production, farming and logging. Most people in Bugala Island live in clusters around fishing villages and trading centres. Public health indicators in the district are poor: 44% of households are without access to safe water, only 31% of pregnant women attend the fourth antenatal care visit and less than 20% of expected pregnant women deliver in health facilities [30,31]. The HIV prevalence in the region is higher than the national average and is estimated to be upwards of 25% [32]. The large burden of HIV is at least in part due to engaging in high-risk behaviour including commercial sex, and the extremely poor status of the local health system. Furthermore, access to HIV testing is severely limited on Bugala Island, as there is only one HIV voluntary counselling and testing (VCT) centre.

The island has 11 health facilities licensed by the Uganda Medical and Dental Practitioners’ Council for doctors’ clinics, nursing homes and hospitals. These facilities are ranked from 1 to 4 according to care level, with 4 indicating comprehensive service provision. The facilities are either public, faith based or private. Private facilities operate on a fee for service model. Faith-based facilities offer subsidies and often charge a co-pay. Government facilities are free but are often short of health workers, medicines and supplies, and patients often have to seek care or buy medicines in private facilities. Only two health facilities on the northern part of Bugala Island provide ART services, both of which are ranked as the highest tiered facilities on the island. Four of the 11 health facilities were excluded from the study as they are located on the southern part of the island and were not accessed by any of the surveyed population in the study. This study was reviewed and approved by the Institutional Review Boards of the University of Washington; the School of Public Health, Makerere University, Uganda; and the Uganda National Council for Science and Technology.

### Household sampling

We conducted household surveys in August, 2012, among a cross-sectional sample of 447 heads of household from residences located in communities from the northern portion of Bugala Island (00°18′32″S, 32°13′30″E). Heads of household were selected using a two-stage cluster sampling scheme. In the first sampling stage, 35 to 45 villages (local council units) on the northern portion of Bugala Island were randomly selected using simple random sampling. The sampling frame was provided by district authorities and a random number generator was used. In the second sampling stage, an approximate map of the distribution of households in the selected villages was generated and a non-probability random sample of households was selected for participation in the study as follows: a randomly selected household in each of the selected village was chosen as the starting point of the survey, and every subsequent 10th household from that point was surveyed until the required sample size was achieved. Inclusion criteria included self-reported head of household, 18 years of age or older, and willing and able to provide informed consent.

### Healthcare access

Access to, and uptake of, healthcare on Bugala Island was defined as reporting uptake of any form of healthcare on Bugala Island in the last five years at any health facility. The specified facility was assumed to be the health facility where healthcare was most commonly sought or where the individual would most likely go at the time of the survey. Responses ranged widely from informal drug shops to licensed health facilities. Global Positioning System (GPS) data and health-facility-level characteristics, including HIV services provided, were captured on the 11 licensed health facilities on Bugala Island.

### Spatial analysis

Straight-line (Euclidean) distance was calculated between each residence and the health facility accessed, as well as between each residence and the nearest health facility, using Spatial Analyst in ArcGIS 10.1 [9]. Euclidean distance between target population and closest service provider is often used to approximate the effort (in both time and cost) needed to access nearby services. This approach has been used in a variety of settings to evaluate the accessibility of healthcare services, from primary care [33] to antenatal care [34] and health services in general [35]. Despite its limitations [36], Euclidean distance is considered a valid, albeit crude, measure of accessibility in both urban and rural settings [33,37] and provides an accurate measure of perceived healthcare accessibility compared with more complex time and cost-based GIS models [38]. In addition to Euclidean distance, we also used self-reported time and cost of travel to access healthcare as alternate measures of accessibility. Travel time and cost are considered more accurate measurements of healthcare accessibility in some settings [39], although they are prone to measurement error when ascertained by self-report. Locational data were geo-rectified to the Universal Transverse Mercator (UTM) zone 36 N projection, 1984 datum. Maps were generated and visualized in ArcGIS 10.1 [40].

### Statistical analysis

We compared the following outcomes between those who reported positive versus negative HIV status for our primary analysis:

1. Mean Euclidean distance to health facility accessed
2. Mean travel time to health facility accessed
3. Mean cost of travel to health facility accessed
4. Probability of accessing the closest health facility

Among those who self-reported positive HIV status, we compared the following metric by residential distance to the nearest ART facility:

1) Probability of accessing a facility that provides ART

Our primary analysis was restricted to individuals with self-reported HIV status (positive or negative), who reported having received healthcare on Bugala Island in the last five years and for whom GPS data on household location were available. Multiple linear regression was used to compare metrics of healthcare access between those with positive self-reported HIV status and those with negative self-reported HIV status, adjusting for age, occupation, income and availability of nearby services (measured as the distance from residence to the nearest documented health facility). Relative risk regression using log binomial models with robust standard errors was used to estimate the adjusted relative risk (aRR) of seeking healthcare at the nearest licensed health facility between those reporting positive HIV status and those reporting negative status, with further analysis stratifying the availability of ART services at the nearest facility. ART was only available at two of the seven licensed health facilities where individuals in our sample went for care. We tested whether PLHIV tend to uptake ART services at a facility with ART/HIV services closest to their residence. We also used log binomial relative risk regression to test the hypothesis that, among PLHIV, the uptake of care at a health facility that provides ART services declines with increasing geographic distance from residence. All models were adjusted *a priori* for age, occupation, income and distance to the nearest facility, an indicator of residential proximity to care.

## Results

**Figure 1:**
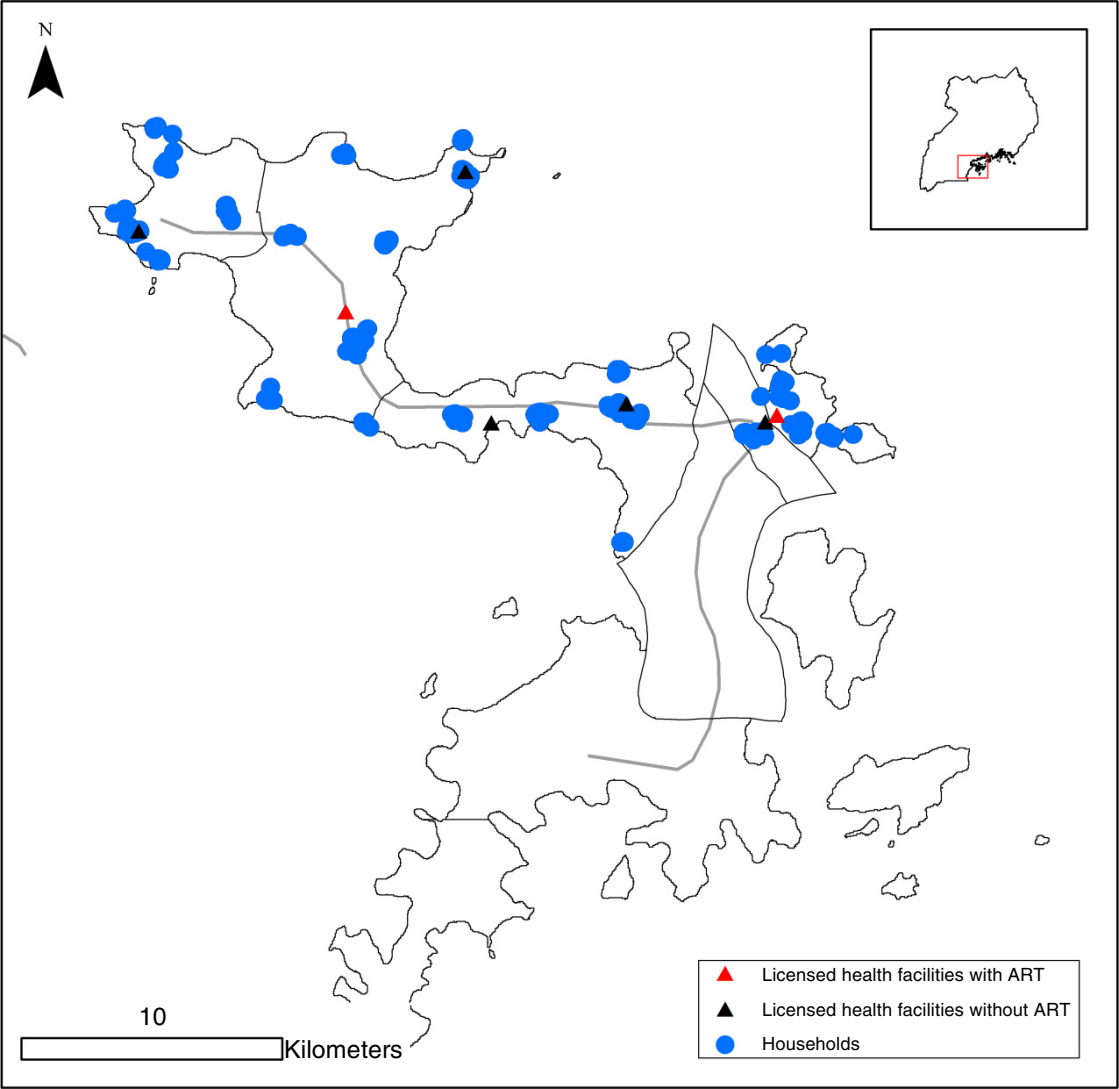
Spatial distribution of 447 heads of household and seven official health facilities surveyed (ART services indicated) on the northern portion of Bugala Island, Uganda.

A total of 447 heads of household were surveyed at their residence on the northern portion of Bugala Island. The population distribution of those surveyed is shown in Figure 1 with the locations of seven licensed health facilities accessed overlaid and classified according to whether or not the facility provides ART services. Those who sought care off the island (*N*=30) were disproportionately HIV positive (40.0%) compared with those who sought care on the island (26.1%).

Individuals were excluded for the following reasons: one for missing GPS data, thirty for seeking care at health facilities located *off* Bugala Island, three for not reporting seeking care at all, two for not reporting where they sought care and thirty-two for missing HIV status, leaving *N*=379 for the primary analysis. The maximum distance from residence to the nearest health facility among those included was 5.3 km, with a median distance of 1.3 km (IQR=0.70 to 3.1 km). Motorcycle (*bodaboda*) was the most common form of transportation to access healthcare (61.2%), followed by walking (33.3%). Median time to access healthcare was 30 minutes (IQR=15 to 45 minutes), and median cost of transport to access healthcare was $1.10 USD (IQR=0 to $2.20 USD).

The majority of those who reported having accessed healthcare on Bugala Island sought care from one of seven licensed health facilities on the northerner part of Bugala Island (94.2%). Among those individuals, the maximum distance from household to health facility accessed was 26.3 km, with a median distance of 3.6 km (IQR=1.3 to 6.9 km). The remaining 5.8% who did not seek care at a licensed health facility reported having accessed healthcare at an informal health facility or drug shop, which could not be geo-located.

The self-reported prevalence of HIV was 26.1% (95% CI (21.7 to 30.6%)) among those included in the sample. Table 1 shows the distribution of demographic and healthcare access variables by self-reported HIV status. Compared with those reporting HIV-negative status, PLHIV were older (mean age 36.4 years vs. 33.7 years, *p*=0.05), more likely not to have completed any formal education (20.4% vs. 5.8%, *p*<0.001), less likely to be in a monogamous marriage (46.9% vs. 64.2%, *p*=0.002), more likely to be a widow/widower (13.3% vs. 5.0%, *p*=0.006) and more likely to work as a fisherman/fisherwoman (13.1% vs. 6.1%, *p*=0.02) or fish seller (17.2% vs. 9.6%, *p*=0.04). No differences in gender or mode of transportation were observed between these groups.

**Table 1:** Characteristics of heads of household reporting positive versus negative HIV status on Bugala Island, Uganda, 2012

	HIV-positive count (%) (*N*=99)	HIV-negative count (%) (*N*=280)
Age (years)		
18 to 24**	7 (7.1)	64 (22.9)
25 to 39	58 (58.6)	147 (52.7)
40 +	34 (34.3)	68 (24.4)
Sex (% female)	59 (64.8)	177 (67.6)
Monthly household income ($USD)		
< 15	35 (37.2)	84 (30.9)
15 to 29	28 (29.8)	73 (26.8)
30 +	31 (33.0)	115 (42.3)
Number of individuals in household		
< 5	73 (73.7)	182 (65.2)
5 to 9	23 (23.2)	90 (32.3)
10 +	3 (3.0)	7 (2.5)
Highest level of education completed		
None**	20 (20.4)	16 (5.8)
Primary	62 (63.3)	172 (62.3)
Secondary**	16 (16.3)	78 (28.3)
College/university	0 (0.0)	10 (3.6)
Marital status		
Married (monogamous)*	46 (46.9)	179 (64.2)
Married (polygamous)	16 (16.3)	34 (12.2)
Separated	17 (17.4)	33 (11.8)
Single (never married)	1 (1.0)	14 (5.0)
Widow/widower*	13 (13.3)	14 (5.0)
Other^a^	5 (5.1)	5 (1.79)
Occupation		
Works in a bar	7 (7.1)	10 (3.6)
Self-employed/small business	8 (8.1)	32 (11.4)
Housewife	12 (12.1)	43 (15.4)
Fisherman/woman*	13 (13.1)	17 (6.1)
Fish seller*	17 (17.2)	27 (9.6)
Farmer*	16 (16.2)	76 (27.2)
Other^b^	26 (26.3)	71 (25.4)
None	0 (0.0)	4 (1.4)
Mode of transportation		
Motorcycle	64 (64.7)	168 (60.0)
Walk	29 (29.3)	97 (34.6)
Bicycle	0 (0.0)	10 (3.6)
Car/truck	2 (2.0)	2 (0.71)
Mini bus or other public transport	4 (4.0)	3 (1.1)
Distance to nearest licensed health facility (km)		
< 1	26 (26.3)	85 (30.4)
1 to 3*	52 (52.5)	113 (40.4)
3 +	21 (21.2)	82 (29.3)
Distance to licensed facility accessed (km)^c^		
< 1	5 (5.3)	29 (11.1)
1 to 3	27 (28.4)	87 (33.2)
3 +	63 (66.3)	146 (55.7)

Proportions calculated out of all non-missing responses among those individuals with non-missing GPS data (*N*=379);

a Includes “steady girlfriend,” “cohabitating” or unspecified;

b includes reported professions that did not correspond to one of the occupation groups. These professions include nurses, students, food vendors, casual labourers, shop attendants and restaurant staff, among others;

c among those who reported having accessed a licensed facility (*N*=359);

* *p*<0.05;

** *p*<0.001.

PLHIV travelled an average of 1.92 km farther (95% CI (0.63 to 3.21 km), *p*=0.004) than HIV-negative individuals to access healthcare, adjusting for residential distance from the nearest health facility, age, occupation and income. The cost and time to travel to health facility were similar among HIV-positive individuals (difference in mean cost 0.46 USD (95% CI 0.03 USD less to 0.94 USD more) and HIV-negative individuals (difference in mean time 5.21 minutes (95% CI 4.24 minutes shorter to 14.65 minutes longer) (Table 2).

**Table 2:** Comparison of healthcare access by HIV status

	Population	HIV-positive count (%/mean)	HIV-negative count (%/mean)	aRR/difference in means (95% CI)^a^	*p*
Healthcare accessibility					
Cost of travel to facility ($USD)^b^	Full sample (*N*=379, 99 HIV+, 290 HIV −)	98 (1.96)	277 (1.49)	0.46 (−0.03 to 0.94)^d^	0.066
Time of travel to facility (minutes)	Full sample (*N*=379, 99 HIV+, 290 HIV −)	99 (41.3)	277 (41.0)	5.21 (−4.24 to 14.65)^d^	0.279
Distance to facility accessed (kilometre)	Accessed a licensed health facility (*N*=357, 95 HIV+, 262 HIV −)	95 (6.13)	262 (4.28)	1.92 (0.63 to 3.21)	**0.004**
Health-seeking behaviour					
Accessed facility is nearest to residence^c^	Nearest facility lacks ART (*N*=215, 59 HIV+, 156 HIV −)	10 (17.9)	50 (34.5)	0.44 (0.24 to 0.83)	**0.011**
	Nearest facility supplies ART (*N*=164, 40 HIV+, 124 HIV −)	35 (89.7)	111 (94.9)	0.95 (0.86 to 1.05)	0.328
Accessed facility that supplies ART^c^	Accessed a licensed health facility (*N*=357, 95 HIV+, 262 HIV −)	80 (80.8)	190 (67.4)	1.26 (1.11 to 1.43)	**<0.001**

Means and proportions calculated out of all non-missing responses;

a adjusted for distance to nearest licensed health facility, age, occupation and income;

b two individuals with extremely outlying one-way costs of travel (>$15) over a short distance by hired motorcycle were changed to missing as these values are likely due to data recording errors;

c adjusted additionally for distance to nearest licensed health facility providing ART services to control for the availability of nearby facilities that supply ART and other higher level care;

d negative value indicates larger cost/travel time among HIV-negative relative to HIV-positive individuals. Bold values indicate significance at α<0.05.

PLHIV differed from HIV-negative individuals with respect to where they sought care on Bugala Island. PLHIV were less likely to access healthcare at the nearest health facility to their residence than HIV-negative individuals (aRR=0.76, 95% CI (0.60 to 0.96), *p*=0.02), adjusting for distance to nearest health facility, age, occupation and income (Table 2). This association, however, was only observed among those whose nearest health facility to their household lacked ART services (among those whose nearest health facility lacked ART services aRR= 0.44, 95% CI (0.24 to 0.83), *p*=0.011; among those whose nearest facility supplied ART services aRR=0.95, 95% CI (0.86, 1.05), *p*=0.328), adjusting for the same variables. As such, PLHIV were more likely to access healthcare from health facilities that provided ART services (aRR=1.26, 95% CI (1.11 to 1.43), *p*<0.001). Among those individuals who sought care at one of two facilities that provide ART (see Figure 1 for geographic locations of facilities), those with self-reported HIV-positive status (*N*=80) went an average of 2.2 km further for healthcare than those with self-reported HIV-negative status (*N*=190) (6.8 km vs. 4.6 km, respectively, *p*=0.001). This difference may be due to a preference among PLHIV for Kalangala Hospital, the highest tiered facility that provided ART services. All individuals whose closest facility was Kalangala Hospital reported accessing healthcare there. By contrast, among those whose closest facility was the lower tiered facility providing ART, 30% of PLHIV and 16% of HIV-negative individuals reported travelling further to access healthcare at Kalangala Hospital (aRR=1.91, 95% CI (1.00 to 3.65), *p*=0.05).

Among PLHIV (*N*=99), 15% reported having accessed healthcare at a facility that did *not* provide ART services. Furthermore, the probability that PLHIV accessed healthcare at a facility providing ART services was 7.1% lower [95% CI 3.6% to 10.4%, *p*<0.001] for every additional kilometre in residential distance from the two facilities that provided ART, adjusting for age, occupation and income, with the largest decrease in uptake at distances over 2 km in residential distance, after which the relationship declines minimally (Table 3).

**Table 3:** Association between access to a facility providing ART and distance to the nearest facility providing those services among *N*=99 PLHIV

Distance band (km)	aRR^a^	95% CI	*p*
0 to 2	1.00	Ref.	–
3 to 5	0.78	0.61 to 0.99	0.044
6 to 10	0.71	0.58 to 0.87	0.001

a Adjusted for age, occupation and income.

## Discussion

In this study, we quantify the unique challenges PLHIV face when accessing healthcare in order to inform how health services interventions might optimize access to, and uptake of, specialized HIV services among PLHIV. We found that PLHIV travel farther and pay more to access healthcare compared with HIV-negative individuals residing in the same communities. PLHIV are less likely to access healthcare from the closest facility to their residence, most likely out of the need for specialized HIV services, such as ART, which are only available at a limited number of facilities located in city centres. We also found that PLHIV are less likely to seek healthcare at facilities with ART services, the farther they live from those facilities. In rural areas with limited access to healthcare services, interventions that aim to increase the distribution and availability of HIV services may increase their uptake among PLHIV.

Our results are consistent with other studies documenting the challenges PLHIV in sub-Saharan Africa face when accessing healthcare [2,15–17,26,41–44]. Many studies have found high transportation costs as one of the most consistent barriers PLHIV face when accessing HIV care [4,15]. Geographic distance is associated specifically with low uptake of ART in developing countries [2]. HIV stigma is also a major barrier to the uptake of specialized HIV services [15,22,42,45]. PLHIV chose not to seek healthcare in some areas due to fear of social exclusion should their HIV status become known in their communities [5,46]. There is some evidence to suggest that PLHIV intentionally bypass the nearest heath facility out of such fear of stigmatization from members of their own communities [24]. While we did not directly measure the effect of stigma on health-seeking behaviours, the fact that PLHIV were just as likely as HIV-negative individuals to seek healthcare at the closest facility to their residence, so long as that facility provided ART services, indirectly suggests that stigma was an unlikely factor in explaining why PLHIV travel farther for healthcare in this population. Further study is needed to confirm this observation.

Our results are also consistent with other studies showing that individuals with conditions that require specialized care will go farther for healthcare compared with those who have more minor syndromes or less specialized needs [47]. In our study and in others comparing health-seeking behaviours between those with and without specialized needs, distance from residence to health facility is less of a factor in uptake of care or determination of where to seek care for those with more severe or specialized conditions [47,48]. Furthermore, our study is consistent with others indicating that people will travel farther to access more advanced services [14,48–50]. This phenomenon is consistent with the idea that those with more pressing needs for healthcare will be more likely to overcome barriers to access healthcare given the urgency of their healthcare need. Still, we found distance to be a determinant in access to ART services among PLHIV, indicating that removing geographic barriers to healthcare may improve their uptake among PLHIV.

A major strength of our study is its ability to demonstrate the additional effort PLHIV expend when seeking healthcare as a result of their HIV-positive status compared with individuals not living with HIV in the same communities. While previous studies have demonstrated a large social and economic burden associated with uptake of healthcare within populations of PLHIV, they have not necessarily shown those barriers to be specific to PLHIV. To overcome this limitation, we chose to compare health-seeking patterns of PLHIV to those of HIV-negative individuals residing within the same communities. In this way, we were able to quantify the additional burden PLHIV face when accessing healthcare, beyond the expected barriers of time, cost and distance associated with accessing healthcare in a rural area in general. Likewise, we highlight the unique challenges PLHIV face when accessing healthcare.

Our sample population was restricted to individuals with a history of direct linkage to the local healthcare system (almost 95% of the study population sought care at a licensed health facility over the past five years) and, as such, does not capture the extent to which certain barriers limit access to healthcare overall. This population does, however, reflect the economic and physical constraints associated with accessing healthcare, which can be used to guide interventions that aim to optimize healthcare access among a more mobilized population of PLHIV. This population consists of those who have already overcome social and economic barriers associated with accessing healthcare. PLHIV who have already linked to care experience lower levels of internalizing HIV stigma, for example [22], which may either be a cause of linking to care or a result (e.g. through uptake of counselling and support systems provided at health facilities) [4].

The results of our study must be considered in the light of certain methodological limitations. First, we were unable to enumerate a comprehensive list of household locations on the island and relied on local administrators to provide approximate maps of where individuals’ households are. Thus, our sample may not fully capture the geographic distribution of the true population. Second, HIV status was captured via self-report and not by laboratory or clinical reports. This method may produce some bias in ascertaining accurate data on HIV status. However, because the study aimed to measure how HIV status impacts healthcare-seeking habits and access to healthcare or treatment, the participants’ knowledge/belief of their own status may be a more relevant factor in influencing healthcare seeking than a laboratory-confirmed diagnosis. Third, we did not collect data on how decisions were made regarding where individuals seek healthcare. To this end, while our results fit the hypothesis that individuals with HIV must go farther to access health facilities with specialized services, we are unable to validate this hypothesis with qualitative, individual-level data to confirm why people chose healthcare at one facility over other available facilities. This limitation may result in some degree of outcome misclassification, especially if an individual choses one health facility for routine healthcare and another for specialized HIV services. Household composition variables were also not collected, precluding the use of indicators such as number of household wage earners or age composition in our analysis. Finally, we did not consider spatial clustering of households in the analyses; instead, we considered information reported from each respondent as independent data with respect to residential location.

Our study's results have important implications for interventions targeted at increasing the access to, and availability of, specialized HIV services in rural areas. PLHIV and their families already face a disproportionate financial burden due to lost work time and increased medical costs associated with the illness. This calls for a re-examination of where to allocate non-invasive HIV services in areas with a limited availability of those services so that more health facilities are able to provide HIV services. In high-burden areas of sub-Saharan Africa with limited availability of HIV services, there is need to increase the provision of ART and other specialized HIV services by strengthening community-based care and treatment systems. Potential strategies to improve access include decentralization of HIV care and treatment to lower-level, community and home-based facilities versus facility-based ART management, task shifting of care to mid-level and lower-level providers, and mobile clinics [51]. Engagement in care and ART adherence are essential to improving clinical outcomes and preventing morbidity and mortality for PLHIV. Our study further emphasizes the importance of strengthening systems to increase the access to, and availability of, specialized HIV services in rural areas in sub-Saharan Africa.

## Conclusions

Our study results indicate that PLHIV travel longer distances to access specialized services. Distance may be a barrier to accessing certain critical HIV services like ART. Increasing the availability of, and access to, specialized HIV services will likely ease the economic and geographic burden incurred on PLHIV seeking healthcare and may even increase the number of individuals linking to such care.
